# PED/PEA-15 Inhibits Hydrogen Peroxide-Induced Apoptosis in Ins-1E Pancreatic Beta-Cells via PLD-1

**DOI:** 10.1371/journal.pone.0113655

**Published:** 2014-12-09

**Authors:** Francesca Fiory, Luca Parrillo, Gregory Alexander Raciti, Federica Zatterale, Cecilia Nigro, Paola Mirra, Roberta Falco, Luca Ulianich, Bruno Di Jeso, Pietro Formisano, Claudia Miele, Francesco Beguinot

**Affiliations:** 1 Dipartimento di Scienze Mediche e Traslazionali dell'Università di Napoli “Federico II”, Naples, Italy; 2 URT dell'Istituto di Endocrinologia e Oncologia Sperimentale Gaetano Salvatore, Consiglio Nazionale delle Ricerche, Naples, Italy; 3 Dipartimento di Scienze e Tecnologie Biologiche e Ambientali, Università del Salento, Lecce, Italy; University of Catanzaro Magna Graecia, Italy

## Abstract

The small scaffold protein PED/PEA-15 is involved in several different physiologic and pathologic processes, such as cell proliferation and survival, diabetes and cancer. PED/PEA-15 exerts an anti-apoptotic function due to its ability to interfere with both extrinsic and intrinsic apoptotic pathways in different cell types. Recent evidence shows that mice overexpressing PED/PEA-15 present larger pancreatic islets and increased beta-cells mass. In the present work we investigated PED/PEA-15 role in hydrogen peroxide-induced apoptosis in Ins-1E beta-cells. In pancreatic islets isolated from Tg_PED/PEA-15_ mice hydrogen peroxide-induced DNA fragmentation was lower compared to WT islets. TUNEL analysis showed that PED/PEA-15 overexpression increases the viability of Ins-1E beta-cells and enhances their resistance to apoptosis induced by hydrogen peroxide exposure. The activity of caspase-3 and the cleavage of PARP-1 were markedly reduced in Ins-1E cells overexpressing PED/PEA-15 (Ins-1E_PED/PEA-15_). In parallel, we observed a decrease of the mRNA levels of pro-apoptotic genes Bcl-xS and Bad. In contrast, the expression of the anti-apoptotic gene Bcl-xL was enhanced. Accordingly, DNA fragmentation was higher in control cells compared to Ins-1E_PED/PEA-15_ cells. Interestingly, the preincubation with propranolol, an inhibitor of the pathway of PLD-1, a known interactor of PED/PEA-15, responsible for its deleterious effects on glucose tolerance, abolishes the antiapoptotic effects of PED/PEA-15 overexpression in Ins-1E beta-cells. The same results have been obtained by inhibiting PED/PEA-15 interaction with PLD-1 in Ins-1E_PED/PEA-15_. These results show that PED/PEA-15 overexpression is sufficient to block hydrogen peroxide-induced apoptosis in Ins-1E cells through a PLD-1 mediated mechanism.

## Introduction

PED/PEA-15 is a small cytosolic protein highly conserved among mammals, ubiquitously expressed [1] and involved in the regulation of several cellular functions, including glucose metabolism, cell proliferation, apoptosis and survival [2]. It features an N-terminal death effector domain (DED) and a C-terminal tail with irregular structure. PED/PEA-15 lacks enzymatic activity and mainly serves as a molecular adaptor [3]. Evidence in the literature shows that PED/PEA-15 is a scaffold protein, modulating signalling pathways relevant to many human diseases such as cancer and Type 2 diabetes [4], [5]. Its expression is increased in different tumors, including human glioma and mammary carcinomas [6], [7] and in several tumor cell lines derived from human larynx, cervix and skin tumors [8], [9]. PED/PEA-15 functions either as a tumor-promoter or as a tumor-suppressor, regulating both proliferation and apoptosis [10]. Cell proliferation can be blocked by PED/PEA-15 binding to ERK. This interaction sequesters ERK in the cytosol, preventing the phosphorylation of its nuclear substrates [11]. On the other hand, PED/PEA-15 modulates tumor cell survival and contributes to resistance to chemotherapy, interfering with apoptotic pathways [9]. PED/PEA-15 antiapoptotic action has been investigated in several cell types and has been shown to interfere with both the extrinsic and intrinsic apoptotic pathways through several distinct mechanisms. Indeed, PED/PEA-15 can reduce the stress-induced apoptosis caused by serum deprivation and oxidative stress, decreasing signalling through the stress-activated protein kinases JNK and p38 [12]. Moreover, PED/PEA-15 interferes with apoptotic mechanisms triggered upon the release of proapoptotic mitochondrial proteins into the cytoplasm, preventing the degradation of the antiapoptotic protein XIAP [13]. PED/PEA-15 further regulates apoptosis by blocking death signaling involving members of the Tumor Necrosis Factor (TNF) receptor superfamily triggered by FASL, TNF alpha, and the Tumor Necrosis Factor-related apoptosis-inducing ligand (TRAIL) [4], [6]. Finally, it has been described that PED/PEA-15 antiapoptotic function is due to the binding of its DED domain to others DED-containing proteins, including Fas-associated protein with death domain (FADD), FADD-like IL-1β-converting enzyme (FLICE), and procaspase-8. This interaction leads to the recruitment of PED/PEA-15 to the Death-Inducing Signalling Complex (DISC) and competitively inhibits the binding of DED containing proteins to the initiator caspases [4]. Several studies have recently shown a key role for PED/PEA-15 in Type 2 diabetes. PED/PEA-15 is known to be overexpressed in adipose and skeletal muscle tissues and in skin fibroblasts from type 2 diabetic individuals causing resistance to insulin action in glucose uptake [5] through its interaction with phospholipase D-1 (PLD-1) and dysregulation of PKC signalling [14], [15]. Interestingly, beta-cell-specific PED/PEA-15 transgenic (beta-tg) mice show impaired glucose tolerance and abnormalities in basal and glucose-stimulated insulin secretion [16].

The immunohistochemistry and morphometric analysis of islets from these animals evidenced that islets were larger and showed a more elongated and irregular shape despite a normal distribution of alpha- and beta-cells, compared with islets from control animals. Quantitative morphometry also showed a two fold increase of islets and beta-cells mass per unit of total pancreatic area in beta-tg mice without significant difference in their pancreas weight [16]. However, the molecular basis of these alterations have never been elucidated and whether PED/PEA-15 affects the survival of beta-cells remains unknown. It is known that beta-cell mass is regulated by a balance between proliferation/neogenesis and cell death, mostly due to apoptosis [17]. Thus, in the present work we have investigated the effect of PED/PEA-15 on hydrogen peroxide-induced apoptosis in Ins-1E beta-cells. We found that PED/PEA-15 overexpression is sufficient to block the apoptosis caused by hydrogen peroxide exposure, at least in part, through a PLD-1 mediated mechanism.

## Materials and Methods

### Cell lines and transfection

Rat Ins-1E beta-cell line was kindly donated by P. Maechler (University of Geneva). Ins-1E cells cultured in regular RPMI 1640 medium with 11 mM glucose, supplemented with HEPES, 5% fetal calf serum, 50 µM 2-mercaptoethanol, 1 mM pyruvate, and antibiotics, as described in [18]. Culture medium was replaced every two days and cells were split once a week. Cells free of micoplasma contamination were used in all experiments.

The cDNA of PED/PEA-15 cloned in pcDNA3 expression vector as previously described [5] and was transfected into the Ins-1E beta-cells using the lipofectamine (Life Technologies, Inc.) method. G-418 was used at the effective dose of 0.5 mg/ml. Individual G-418-resistant clones were selected by the limiting dilution technique [16]. Ins-1E_CTRL_ and Ins-1E_PED/PEA-15_ cells were treated as indicated with hydrogen peroxide (70 µM) and propranolol (150 µM). Time of incubation and concentration of propranolol were set performing preliminary dose/response sulforhodamine experiments to avoid toxicity (data not shown). The D4 cDNA was amplified by PCR from HeLa cells and cloned into pcDNA3-HA vector as previously described [33]. The construct pcDNA3-HA-D4 was transiently transfected in Ins-1E cells using the lipofectamine reagent according to the manufacturer's instructions. D4 expression levels Ins-1E_CTRL_ and Ins-1E_PED/PEA-15_ cells were evaluated by qualitative PCR using the following human primers:

5′-CGATCAAGCTTCGCTGGAACTTCACAAAAAT-3′

5′-CGATCTCTAGAGGATACGAGAATGCGTCAGG-3′

### Mouse models and pancreatic islets isolation

Tg_PED/PEA-15_ mice [15] were backcrossed for 12 generations on the C57/Bl6 background. 4 months old, male and female Tg_PED/PEA-15_ mice and their non-transgenic littermates (wild type; Wt) were used for all studies. All procedures and euthanasia were conducted in according to the guidelines and approved by the Institutional Animal Care and Utilization Committee (Ministero della Salute, Dipartimento della Sanità Pubblica Veterinaria, della Sicurezza Alimentare e degli Organi Collegiali per la Tutela della Salute, Direzione Generale della Sanità Animale e dei Farmaci Veterinari)

Islets were isolated from 4-month-old C57Bl/6J mice (WT) and Tg_PED/PEA-15_ mice by collagenase digestion, handpicked under a stereomicroscope as described in [20] and cultured in complete RPMI 1640 medium. A total of 25 islets were manually selected, incubated 16 h in presence or absence of 10 µM hydrogen peroxide [21] and apoptosis was quantitatively determined using the Cell Death Detection ELISAPLUS kit (Roche Diagnostics - Mannheim, Germany) according to manufacturer's instructions [6].

### Sulforhodamine assay

The sulforhodamine B (SRB) assay was used as described previously [22]. Briefly, 10^4^ cells in 100 µl of medium/well were plated in 96-well flat-bottomed microtiter plates. 24 h after plating (an adequate time for exponential growth recovery) culture medium was replaced with fresh medium either containing or not containing the hydrogen peroxide. At the end of hydrogen peroxide exposure, cells were fixed with 50% TCA for 2 h at 4°C and stained with 0.4% SRB (Sigma Aldrich) dissolved in 1% acetic acid for 30 minutes. The plates were then washed four times with 1% acetic acid to remove unbound stain, air-dried and solubilized in 100 µl of 10 mM unbuffered Tris base (tris(hydroxymethyl)aminomethane) solution. The optical density of treated cells was detected at 490 nm. Each sample was run in octuplet, and each experiment was repeated three times.

### Cell Death Detection Assay

The mechanism of cell death was quantitatively determined using the Cell Death Detection ELISA^PLUS^ kit (cat. No. 11774425001; Roche Diagnostics - Mannheim, Germany) [6]. The Cell Death Detection ELISA kit is designed for quantitative detection of mono- and oligonucleosomal DNA fragmentation when cells undergo apoptotic death *in vitro*.

After treatments, Ins-1E_PED/PEA-15_ and control cells were processed as described by the manufacturer's instructions. Necrotic cells were removed by centrifugation and the pellet (apoptotic fraction) was analyzed by ELISA. Measurements were made at 405 nm against an ABTS solution as a blank (reference wavelength ∼490 nm) using an Infinite F200 Tecan multimode reader.

### TUNEL assay

For TUNEL staining the In Situ Cell Death fluorescent kit (Roche Diagnostic - Mannheim, Germany) was used, following the manufacturer's instructions. Briefly, Ins-1E_PED/PEA-15_ and control cells were fixed with 4% paraformaldehyde for 1 h and permeabilized in a 0.1% Triton X-100 - 0.1% sodium citrate solution for 2 min on ice. Subsequently, 50 µl of TUNEL reaction mixture was added to each sample and cells were incubated for 1 h at 37°C. Cells were counterstained with DAPI before mounting [23]. Microscopy and imaging were performed in a Zeiss AxionPlan II epifluorescence (FluoArc) Microscope. Images were processed using Axion Vision software and the Image J software.

### Immunofluorescence

Ins-1E_PED/PEA-15_ and control cells plated on coverslips were fixed with 4% paraformaldehyde for 20 min and permeabilized with 0.1% Triton X-100 in PBS for 20 min. Cells were exposed to blocking buffer (2% normal donkey serum in PBS (Jackson ImmunoResearch, Jacksonville, MI) for 1 h and incubated overnight with primary antibody anti-cleaved Caspase-3 (#9661S, Cell Signaling Technologies, Beverly, MA) diluted 1∶400 in blocking buffer. Antigen-bound primary Caspase-3 antibody was detected by 1 h incubation with a 1∶200 dilution of Rhodamin RedX conjugated donkey anti-rabbit secondary antibody (#111-075-003, Jackson Immunolabs, West Grove, PA). All incubation steps were separated by washing in 0.1% Triton X-100 in PBS for 3×5 min [23]. Images were obtained in a Zeiss AttoArc II epifluorescence microscope.

### Western blot analysis

For Western blot analysis, after treatments Ins-1E_PED/PEA-15_ and control cells were solubilized in lysis buffer (50 mM HEPES, pH 7.5, 150 mM NaCl, 10 mM EDTA, 10 mM Na_2_P_2_O_7_, 2 mM Na_3_VO_4_, 100 mM NaF, 10% glycerol, 1% Triton X-100, 1 mM phenylmethylsulphonylfluoride, 10 µg/ml aprotinin) for 2 h at 4°C. Cell lysates were clarified by centrifugation at 5,000 *g* for 20 min, separated by SDS-PAGE, and transferred onto 0.45-µm Immobilon-P membranes (Merck Millipore, Darmstadt, Germany) [24]. Upon incubation with primary PARP (1∶1000; #9542, Cell Signaling Technologies, Beverly, MA), anti-phospho-PKCalpha (1∶1000; #06-822, Merck Millipore, Darmstadt, Germany) and Tubulin (1∶1000, #SC5546, Santa Cruz Biotech Inc) and secondary antibodies (Bio-Rad Laboratories, Inc.), immmunoreactive bands were detected by ECL according to the manufacturer's instructions (Pearce ECL Western Blotting Substrate).

### Reverse Transcriptase and Real-time RT-PCR

Total RNA was isolated from Ins-1E_PED/PEA-15_ and control cells using TRIzol reagent (Life Technologies) according to manufacturer's instructions. After quantification, 1 µg of total RNA was reverse transcribed using Superscript III Reverse Transcriptase (Invitrogen) using random hexamers. Real-time RT-PCR was performed on an iCycler IQ multicolor Real Time PCR Detection System (Bio-Rad Laboratories, Inc) using the comparative CT method (2-^ΔΔCT^) method with a Platinum SYBR Green qPCR Super-UDG mix (Bio-Rad Laboratories, Inc) [16]. All reactions were performed in triplicate, and the relative mRNA expression levels of target genes were normalized to 18S rRNA. Specific primers used for amplification were:

Bcl- x- Fw 5′-3′ AGAGGCTGGCGATGAGTTT

Bcl- xL Rv 5′-3′ AGAAGAAGGCCACAATGCGA

Bcl- xS Rv 5′-3′ GCTGCATTGTTCCCGTAG

BAD – Fw 5′-3′ CCAGAGTTTGAGCCGAGTGAGCA

BAD – Rv 5′-3′ CTGTTATTGGCTGCCTGTCCCG

18s rRNA Fw 5′-3′ GTAACCCGTTGAACCCCATT

18s rRNA Rv 5′-3′ CCATCCAATCGGTAGTAGCG

### Statistical analysis

For statistical analyses, one-way ANOVA followed by Tukey-Kramer multiple comparison test was performed using GraphPad Prism 5.00 (GraphPad Software, San Diego, CA, USA). The statistical significance was set at *p*<0.05. Data are expressed as means ± standard errors of the mean (SEM).

## Results

### Regulation of apoptotic cascade by PED/PEA-15

**Figure 1:**
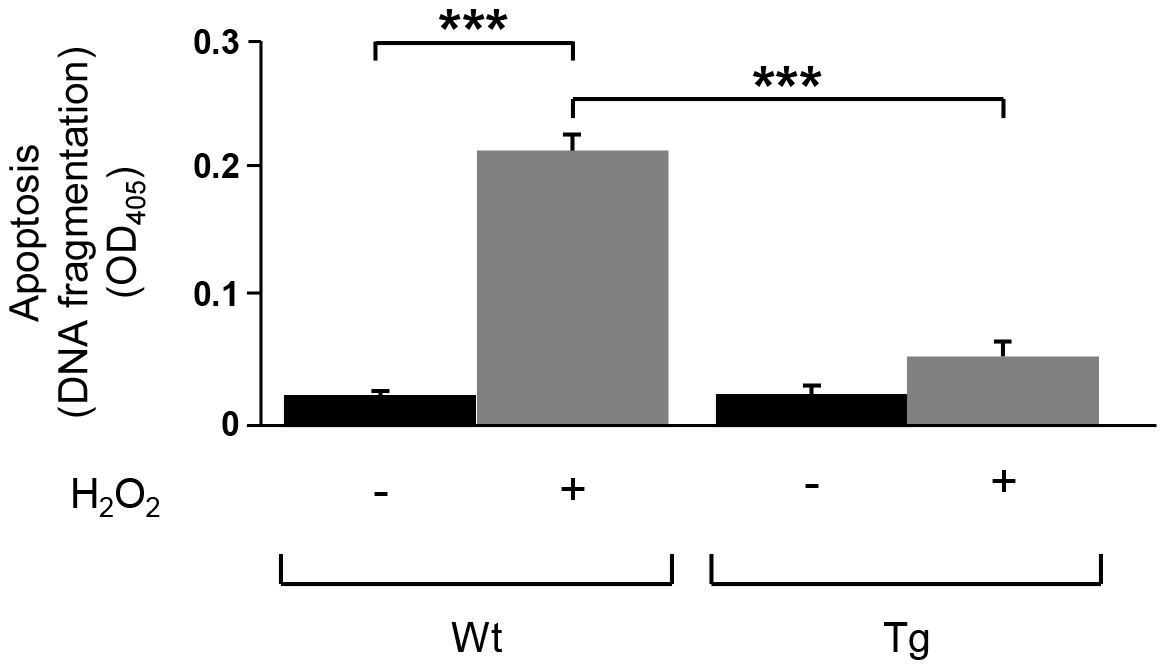
Apoptosis in isolated mice islets. Mouse pancreatic islets were treated or not with 10 µM hydrogen peroxide for 16 hours. After this, histone-associated DNA fragments were quantified by ELISA to evaluate apoptotic cell death. Each column represents the mean ± SE from three separate experiments. ***p<0.001.

To investigate the role of PED/PEA-15 overexpression in beta-cells apoptosis, we isolated pancreatic islets from Tg*_PED/PEA-15_* mice and their non-transgenic littermates (wild type; WT). Islets were incubated 16 h in presence or absence of 10 µM hydrogen peroxide [21] and apoptosis was then quantitatively determined using the Cell Death Detection ELISAPLUS kit. As shown in Fig. 1, in islets from WT mice the treatment with hydrogen peroxide induces a 7-fold increase in apoptosis, compared to untreated islets from the same mice. In contrast, in islets from Tg*_PED/PEA-15_* mice only a 2-fold increase in apoptosis was observed upon hydrogen peroxide incubation.

**Figure 2:**
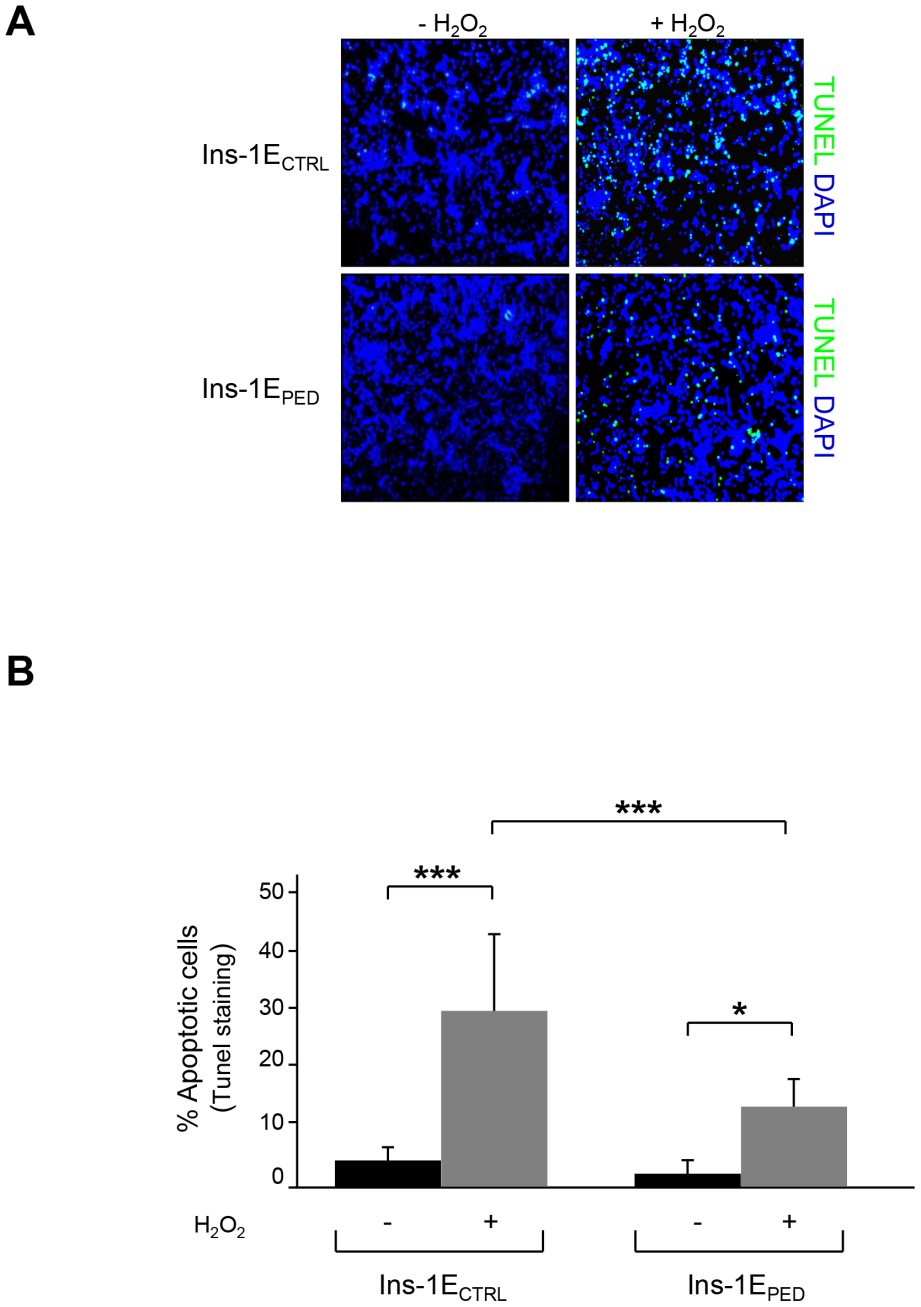
TUNEL analysis. A) Representative microscopic views of apoptosis after the TUNEL staining (image scale 10X). Cells were treated for 48 hours with 70 µM hydrogen peroxide and then stained with TUNEL reagent and DAPI to detect apoptotic (green) and total nuclei (blue), respectively. B) Bar graph represents the quantification of apoptosis by TUNEL assay. Results are quantified as the percentage of TUNEL-positive cells in 4 high magnification fields per slide. Values represent the mean ±SD of three independent experiments. *p<0.05 and ***p<0.001.

**Figure 3:**
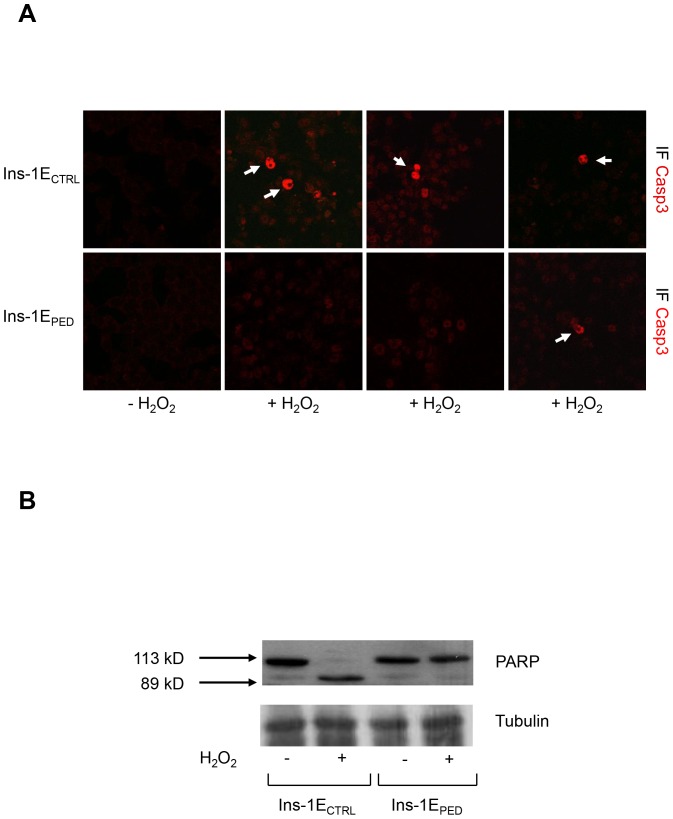
Activation of caspase-3 and PARP cleavage. A) Caspase-3 activation. Cells were grown on a glass coverslips and treated or not with 70 µM hydrogen peroxide for 48 hours. After fixation and permeabilization, cells were processed for anticaspase-3 antibody (in red). Arrows indicate cells stained with antibody to the active form of caspase-3. The total number of cells is visualized by the background staining. The results shown are representative of 3 independent experiments. B) PARP cleavage. Cells were treated or not for 48 hours with 70 µM hydrogen peroxide, as indicated. Whole-cell extracts (30 µg) were analyzed by Western blot with anti-PARP antibody. Full-length PARP (113 kDa) and the cleaved fragment (89 kDa) are indicated by arrowheads. Tubulin was used as loading control (n = 3). A representative image is shown.

To clarify the molecular mechanisms underlying PED/PEA-15 anti-apoptotic action in beta-cells, we then used Ins-1E [18] cells stably transfected with either a PED/PEA-15 full length cDNA (Ins-1E_PED/PEA-15_) or an empty vector (Ins-1E_CTRL_) [16]. Both cell types were treated for 48 h with 70 µM hydrogen peroxide as previously described [25] and TUNEL analysis was performed. TUNEL staining revealed about 12% of apoptotic cells in Ins-1E_PED/PEA-15_ cells exposed to hydrogen peroxide and about 30% of apoptotic cells in Ins-1E_CTRL_ cells (Fig. 2a, 2b). To better characterize the effect of PED/PEA-15 overexpression on the apoptotic cascade, the activation of caspase-3, a downstream marker acting as a final effector of apoptosis [26], was further measured. To this aim, immunofluorescence labelling of cleaved caspase-3 was performed in Ins-1E_PED/PEA-15_ and in Ins-1E_CTRL_ cells upon hydrogen peroxide treatment. As shown in Fig. 3a, the number of cells positive for cleaved active caspase-3 is higher in Ins-1E_CTRL_ than in Ins-1E_PED/PEA-15_ cells upon hydrogen peroxide incubation.

**Figure 4:**
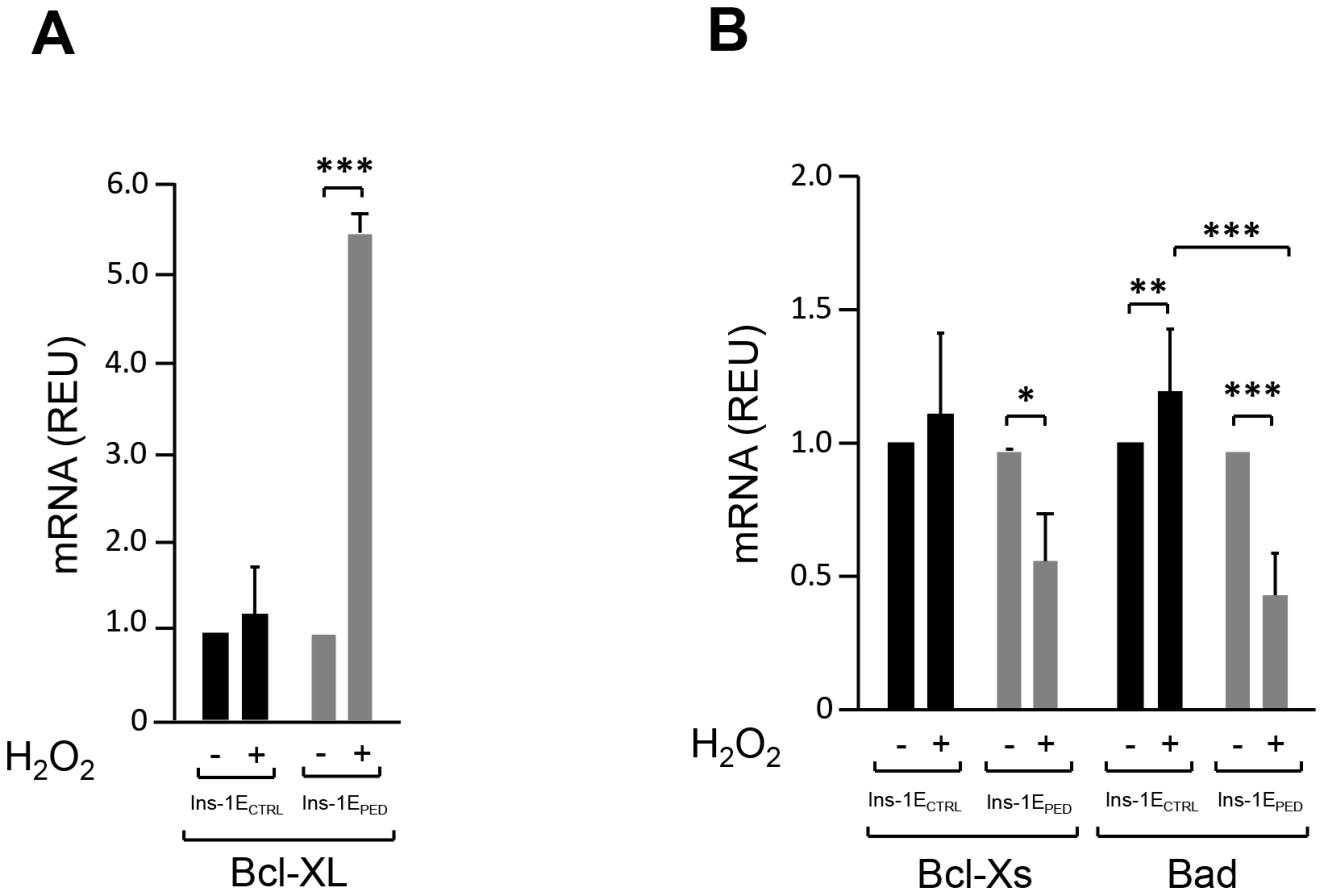
Apoptosis-Related Genes Expression. Cells were treated or not with 70 µM hydrogen peroxide for 48 hours. Total RNA was then isolated and the mRNA levels of Bcl-xL (A), Bcl-xS and Bad (B) genes were assessed by real-time RT-PCR using 18S as internal control. Values represent the mean ±SD of three independent experiments. *p<0.05, **p<0.01 and ***p<0.001.

The cleavage of PARP-1, a well-established substrate for caspase-3 [27], was subsequently analysed. As shown in Fig. 3b, PARP-1 cleavage in response to hydrogen peroxide was detectable in Ins-1E_CTRL_ but not in Ins-1E_PED/PEA-15_ cells. Finally, the expression levels of pro- and anti-apoptotic genes [28] were compared in Ins-1E_PED/PEA-15_ and in Ins-1E_CTRL_ cells upon hydrogen peroxide incubation. Hydrogen peroxide treatment did not induce any significant variation in Bcl-xL mRNA in Ins-1E_CTRL_ cells. In contrast, Bcl-xL expression level in Ins-1E_PED/PEA-15_ cells increased by ∼5-fold upon incubation with hydrogen peroxide compared to untreated cells (Fig. 4a). The abundance of the mRNA of proapoptotic genes such as Bcl-xS and Bad [29] was unchanged in Ins-1E_CTRL_ cells in response to hydrogen peroxide. Interestingly, in Ins-1E_PED/PEA-15_ cells the incubation with hydrogen peroxide reduced the mRNA levels of Bcl-xS and Bad by 43% and 57%, respectively (Fig. 4b). Incubation of Ins-1E_CTRL_ and Ins-1E_PED/PEA-15_ cells with lower concentrations of hydrogen peroxide (30–50 µM) for shorter times (3–8 h) did not significantly modified anti- and pro-apoptotic genes expression (S1 Figure a, b, c).

### Role of PLD-1 in PED/PEA-15 antiapoptotic action

**Figure 5:**
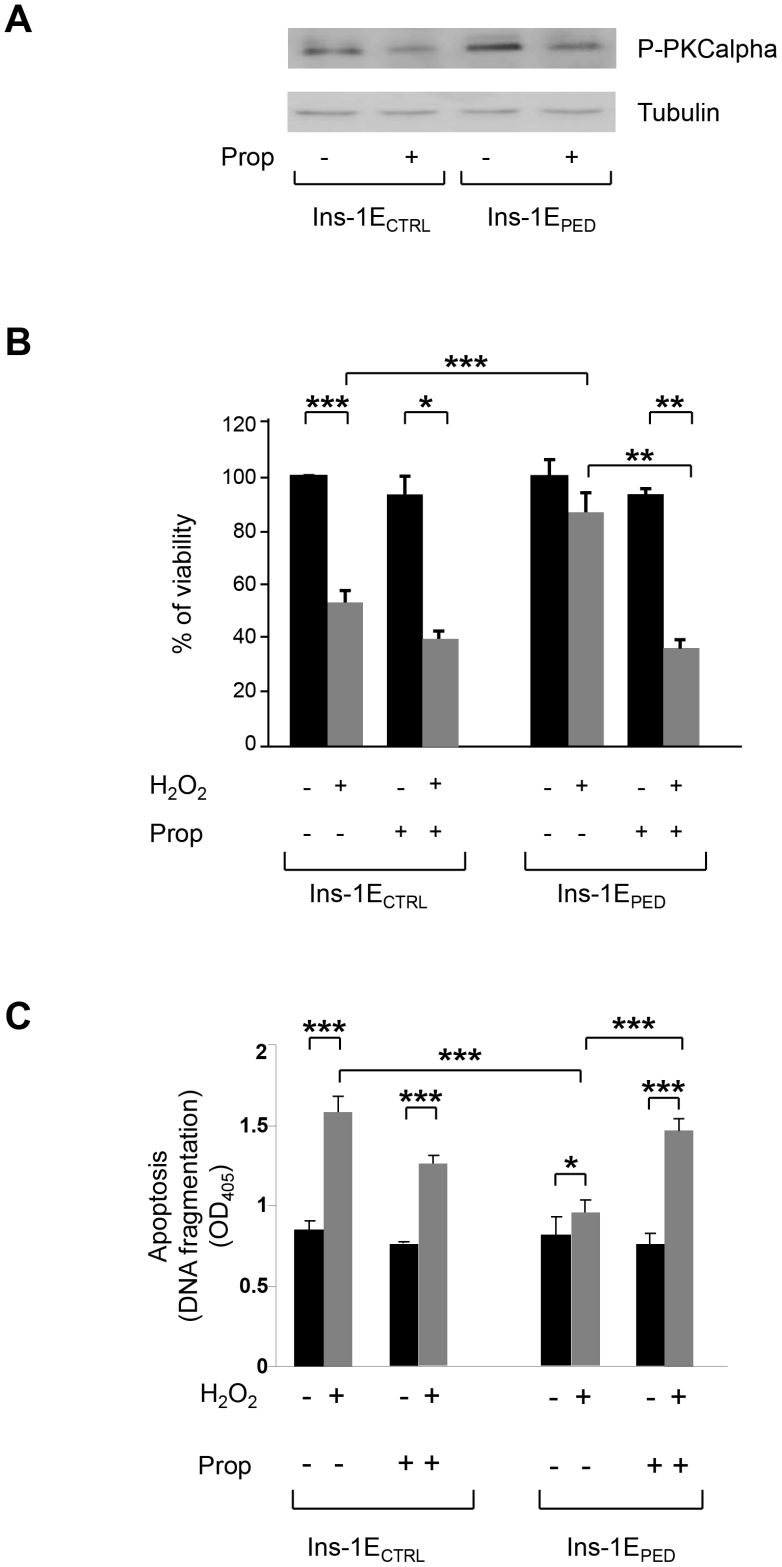
PLD-1 role in PED/PEA-15 anti-apoptotic action. A) PKC alpha phosphorylation. Cells were treated or not for 48 hours with 150 µM propranolol, as indicated. Whole-cell extracts (30 µg) were analyzed by Western blot with anti-phospho PKC alpha antibody. Tubulin was used as loading control (n = 3). A representative image is shown. B) Cell viability. Cells were cultured in 96-well cell culture plates, pretreated with 150 µM propranolol and then incubated with 70 µM hydrogen peroxide as indicated. After 48 hours, cell viability was estimated by use of the sulforhodamine B assay. Cell survival is expressed as the percent of control (untreated Ins-1E_CTRL_ cells). Each point is the mean value from eight identical wells. Values represent the mean ±SD of three independent experiments. *p<0.05, **p<0.01 and ***p<0.001. **C**) Apoptosis. Cells were pretreated with 150 µM propranolol and then incubated with 70 µM hydrogen peroxide as indicated. After 48 hours, cells were harvested and apoptosis was quantitated by evaluating the level of DNA fragmentation using the Roche Cell Death Detection ELISAPLUS. Data are the mean value of four identical wells. Values represent the mean ±SD of three independent experiments. *p<0.05, ***p<0.001.

We have subsequently investigated the involvement of PLD-1 in the antiapoptotic function of PED/PEA-15. To this end, Ins-1E_PED/PEA-15_ and Ins-1E_CTRL_ cells were incubated with 15 µM propranolol, a specific pharmacological inhibitor of PLD-1 pathway [30], before treatment with hydrogen peroxide. As shown in Fig. 5a, PKC alpha phosphorylation is reduced both in Ins-1E_CTRL_ and Ins-1E_PED/PEA-15_ cells incubated with propranolol, compared with untreated cells. Cell viability was then evaluated by sulforhodamine B staining. Fig. 5b showed that viability of Ins-1E_CTRL_ cells is reduced by 50% upon treatment with hydrogen peroxide. Pretreatment with propranolol did not significantly modify cell viability both in the absence and the in presence of hydrogen peroxide. Hydrogen peroxide incubation of Ins-1E_PED/PEA-15_ cells induced a slight (not statistically significant) reduction of viability compared to untreated cells. In contrast, the preincubation of hydrogen peroxide treated Ins-1E_PED/PEA-15_ cells with propranolol reduced cell viability by ∼60%, and almost abolished the apoptosis protective effects conferred by PED/PEA-15 overexpression. Indeed, the viability of Ins-1E_PED/PEA-15_ cells treated with hydrogen peroxide in the presence of propranolol was comparable with that measured in Ins-1E_CTRL_ cells in the same conditions (Fig. 5b).

Next, we measured the effect of PLD-1 inhibition on apoptosis by evaluating the level of DNA fragmentation in Ins-1E_PED/PEA-15_ and in Ins-1E_CTRL_ cells. Hydrogen peroxide incubation increased apoptosis by about 2-fold in Ins-1E_CTRL_ cells and this increase was not significantly modified by pretreatment with propranolol. In contrast, only a 15% increase in apoptosis was observed in Ins-1E_PED/PEA-15_ cells upon hydrogen peroxide treatment (Fig. 5c). Interestingly, the pretreatment with propranolol increases hydrogen peroxide induced apoptosis in Ins-1E_PED/PEA-15_ cells to levels comparable to those observed in Ins-1E_CTRL_ cells (Fig. 5c). Incubation of Ins-1E_CTRL_ and Ins-1E_PED/PEA-15_ cells with lower concentrations of hydrogen peroxide (30–50 µM) for shorter times (3–8 h) did not significantly modified cell viability (S2 Figure a) and DNA fragmentation (S2 Figure b).

**Figure 6:**
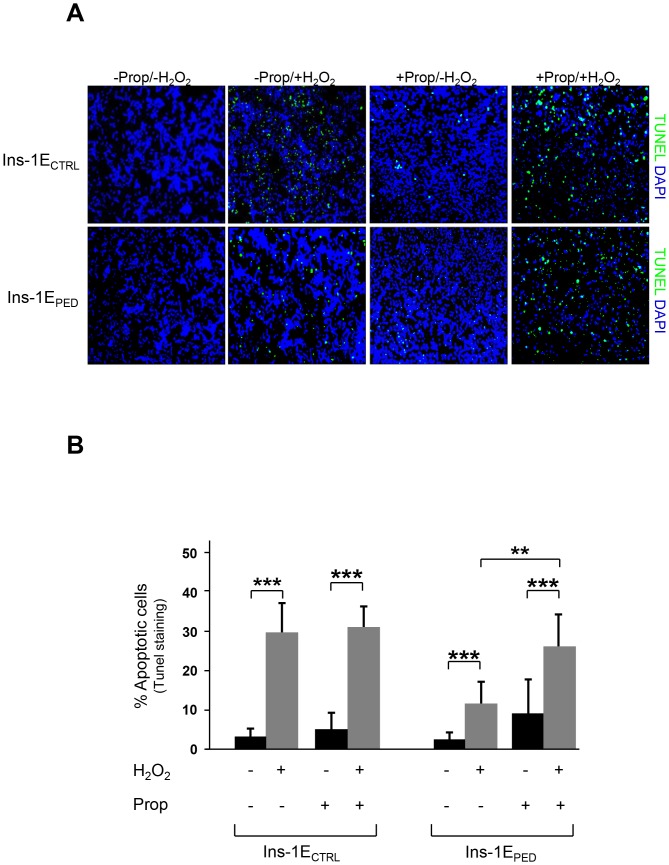
TUNEL analysis. A) Representative microscopic views of apoptosis after the TUNEL staining (image scale 10X). Cells were pretreated with 150 µM propranolol and then treated for 48 hours with 70 µM hydrogen peroxide and then stained with TUNEL reagent and DAPI to detect apoptotic (green) and total nuclei (blue), respectively. B) Bar graph represents the quantification of apoptosis by TUNEL assay. Results are quantified as the percentage of TUNEL-positive cells in 4 high magnification fields per slide. Values represent the mean ±SD of three independent experiments. **p<0.01 and ***p<0.001.

**Figure 7:**
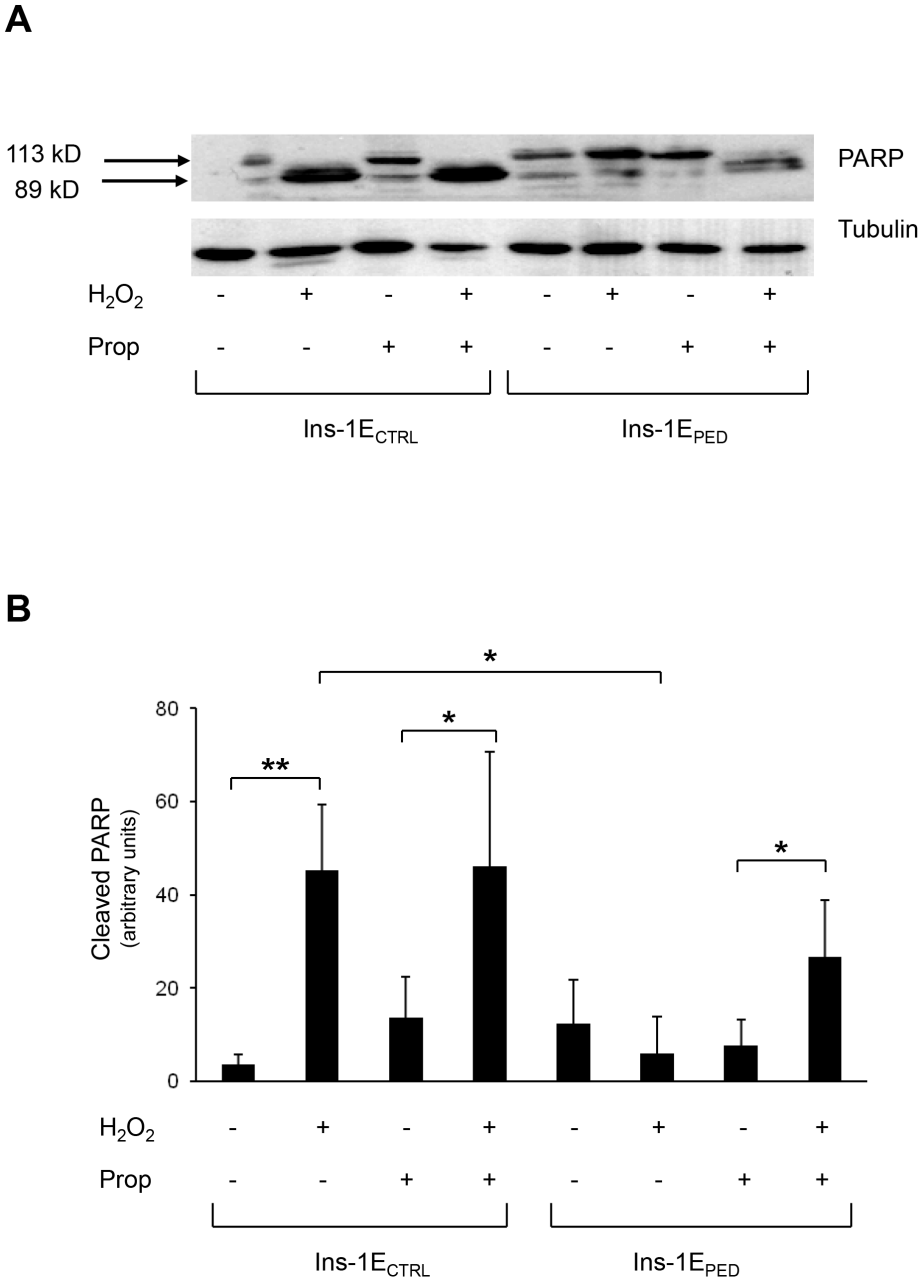
PARP cleavage. **A**) Cells were pretreated with 150 µM propranolol and then treated or not for 48 hours with 70 µM hydrogen peroxide as indicated. Whole-cell extracts (30 µg) were analyzed by Western blot with anti-PARP antibody. Full-length PARP (113 kDa) and the cleaved fragment (89 kDa) are indicated by arrowheads. Tubulin was used as loading control (n = 3). A representative experiment is shown. **B**) The bands for cleaved PARP were scanned, densitometrically quantitated using NIH Image J software and the resulting data were plotted on the bar graph. Values represent the mean ±SD of three independent experiments. *p<0.05, **p<0.01.

TUNEL analysis revealed a 25% increase of apoptosis in hydrogen peroxide treated Ins-1E_CTRL_ cells. Propranolol preincubation did not modify apoptosis in Ins-1E_CTRL_ cells, either in basal condition or in response to hydrogen peroxide. In contrast, in Ins-1E_PED/PEA-15_ PLD-1 inhibition by propranolol reduced the anti-apoptotic action of PED/PEA-15 in presence of hydrogen peroxide (Fig. 6a, 6b). Moreover, PARP-1 cleavage in response to hydrogen peroxide was evaluated by Western blot in propranolol pre-treated cells. Propranolol preincubation did not modify PARP-1 cleavage induced by hydrogen peroxide in Ins-1E_CTRL_ cells. In contrast, in propranolol pre-incubated Ins-1E_PED/PEA-15_ cells PARP-1 cleavage became evident in response to hydrogen peroxide (Fig. 7a, 7b).

**Figure 8:**
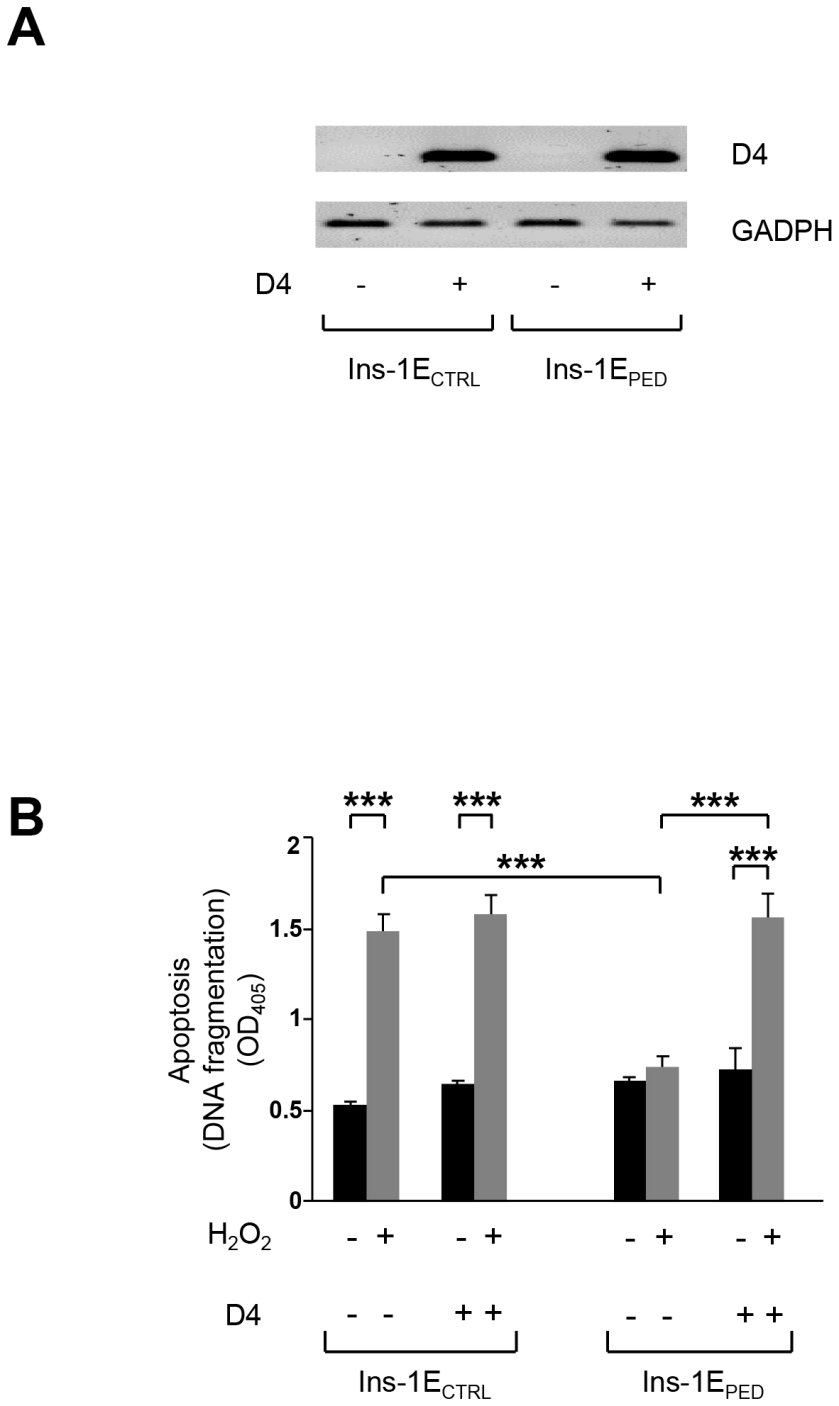
A) *HA-D4 transfection*. pcDNA3-HA-D4 and the control plasmid pcDNA3-HA were transfected in both Ins-1E_PED/PEA-15_ and in Ins-1E_CTRL_ cells, and D4 expression was analyzed by qualitative PCR. **B)** ***Apoptosis.*** Cells were incubated with 70 µM hydrogen peroxide as indicated. After 48 hours, cells were harvested and apoptosis was quantitated by evaluating the level of DNA fragmentation using the Roche Cell Death Detection ELISAPLUS. Data are the mean value of four identical wells. Values represent the mean ±SD of three independent experiments. ***p<0.001.

Then, to investigate the role of PED/PEA-15 interaction with PLD-1 in PED/PEA-15 antiapoptotic action, the HA-tagged D4 domain of PLD-1 (HA-D4), spanning residues 929–1030 [19], was expressed at comparable levels both in Ins-1E_CTRL_ and INS-1E_PED/PEA-15_ cells (Fig. 8a). In Ins-1E_CTRL_ cells, the expression of HA-D4 did not modify hydrogen peroxide induced apoptosis compared to cells transfected with the empty vector. In contrast, in INS-1E_PED/PEA-15_ cells transfected with the empty vector, we observed a slight increase in apoptosis upon hydrogen peroxide treatment. Interestingly, in INS-1E_PED/PEA-15_ cells the expression of HA-D4 increases apoptosis to levels comparable to those occurring in Ins-1E_CTRL_ cells, almost completely blocking PED/PEA-15 antiapoptotic action (Fig. 8b).

## Discussion

PED/PEA-15 is a small scaffold protein, which modulates signalling pathways relevant to several human disorders such as cancer and Type 2 diabetes. In particular, PED/PEA-15 regulates proliferation by binding ERK1/2 and blocks apoptosis by interfering with both the intrinsic and the extrinsic pathways [4], [6], . It has been reported that the selective overexpression of PED/PEA-15 in beta-cells leads to beta-cell hyperplasia and increased beta-cells mass [16]. However, the molecular mechanisms involved in these actions have not been elucidated yet. In the present study we show that the increase in the beta-cell survival might be related to the antiapoptotic activity of PED/PEA-15. Indeed, ELISA showed that DNA fragmentation was higher in islets isolated from Tg*_PED/PEA-15_* mice compared to Wt mice. Moreover, sulforhodamine B staining showed a marked increase in viability of beta-cells overexpressing PED/PEA-15 upon hydrogen peroxide incubation compared to control cells. Both ELISA and TUNEL analysis showed that overexpression of PED/PEA-15 in Ins-1E beta-cell model protects from hydrogen peroxide induced apoptosis. Furthermore, several steps of the apoptotic cascade are modified by the overexpression of PED/PEA-15. Caspase-3 activation, induced by hydrogen peroxide exposure, is lower in Ins-1E_PED/PEA-15_, similar to PARP-1 cleavage. Also the mRNA expression levels of the antiapoptotic gene Bcl-xL in hydrogen peroxide treated Ins-1E_PED/PEA-15_ cells is increased, while the expression of proapoptotic genes such as Bcl-xS and Bad [28], [29] was reduced in Ins-1E_PED/PEA-15_ cells compared to control cells upon hydrogen peroxide incubation. This is not surprising since the balance between pro- and anti-apoptotic members of the Bcl-2 family has been described to modulate apoptosis induced by hydrogen peroxide in several cell type including HeLa cells, neonatal rat cardiac myocytes and PC12 [31]–[33]. In particular, the intracellular amounts of these genes represent a molecular indicator of whether a cell dies or survives. PED/PEA-15 is a known modulator of apoptosis, capable to interfere with both the intrinsic and the extrinsic pathway. However, we cannot exclude that the antiapoptotic effect of PED/PEA-15 could be mediated also by other members of the Bcl-2 family, such as the pro-apoptotic Bim and/or Bid proteins [34], [35], contributing to further disrupt the balance between pro- and anti-apoptotic genes. The molecular mechanism through which PED/PEA-15 can modulate the expression of members of the Bcl-2 family is still unknown. We have previously shown that PED/PEA-15 overexpression in beta-cells inducing the dysregulation of PKC signaling, affects the transcription of genes relevant for beta-cell function, such as Sur1, Kir6.2 and Foxa2 [16]. PED/PEA-15 is a molecular adaptor exerting functions by binding to different interactors, including ERK1/2 [11], Omi/HtrA2 [13] and PLD-1 [14], [15]. The overexpression of PED/PEA-15 does not modify the proliferation rate of Ins-1E beta-cells (S3 Figure). Thus, we hypothesized that PED/PEA-15 does not interfere with ERK1/2 function in this cell type. Since the proapoptotic Omi/HtrA2 mitochondrial serine protease is expressed at very low levels in pancreas [13], we have focused our attention on PLD-1. Previous studies on both cellular and animal models demonstrated that the deleterious effects on glucose homeostasis induced by PED/PEA-15 overexpression are due to its interaction with PLD-1 which, in turn, alters PKC signalling [14], [15], [19]. Pretreatment of Ins-1E with propranolol, a well known inhibitor of PLD-1 pathway, abolishes PED/PEA-15 ability to block hydrogen peroxide-induced apoptosis. This finding suggests that PLD-1 is a key mediator of PED/PEA-15 prosurvival activity in beta-cells. Evidence in the literature shows that PLD-1 is a key regulator of cell survival. Its role in apoptosis is still controversial, since both pro and antiapoptotic effects have been reported in response to different apoptotic stimuli in several cell types [36]. Indeed, overexpression of either PLD-1 or PLD-2 suppress actinomycin D-induced apoptosis [37], due to an increased activation of survival and proliferation signalling pathways involving PI3K, Akt, p70s6k and ERK. Moreover, hydrogen peroxide-induced apoptosis was suppressed by transfection of PLD-2 into PC12 cells [38]. Consistently, in glioma cells, the overexpression of PLD-1 and PLD-2 enhanced the expression of antiapoptotic genes [39]. The overexpression of D4 domain of PLD1, involved in the interaction with PED/PEA-15 [19], almost completely blocked PED/PEA-15 antiapoptotic action. Thus, it is likely that also the antiapoptotic effect of PED/PEA-15 is due to its binding to PLD-1 and to the subsequent stabilization of PLD-1, such as its deleterious effect on glucose homeostasis. Indeed, when transfected into L6_PED/PEA-15_ cells and in myocytes derived from PED/PEA-15-overexpressing transgenic mice, D4 domain abrogates the PED/PEA-15-PLD-1 interaction and reduces protein kinase C-alpha activity to levels similar to controls. Interestingly, the peptide restores insulin-stimulated glucose uptake by approximately 70% [19]. Recent studies have also found that PLD-1 is an upstream regulator of mTOR, a master regulator of beta-cells mass and survival [40]. In particular, the mTORC1 pathway can be regulated by phosphatidic acid [41], a lipid second messenger produced through the hydrolisis of phosphatidylcoline by PLD. We have blocked PLD-1 pathway downstream phosphatidic acid production, using propranolol, which inhibits PLD-1-dependent production of diacylglycerol (DAG) [42]. However, mTOR may not be involved in PED/PEA-15 antiapoptotic action since the levels of apoptosis in response to hydrogen peroxide are higher in propranolol treated Ins-1E_PED/PEA-15_ cells, despite the presence of phosphatidic acid. DAG is a second messenger produced by PLD-1 and activates classical PKC isoforms such as PKC alpha and beta [43]. We have previously shown that the binding of PED/PEA-15 to PLD-1 enhances PLD-1 stability leading to an increase of DAG levels [15]. This, in turn, is responsible for the hyperactivation of PKC alpha and for the deregulation of PKC zeta function in glucose metabolism [15]. Interestingly, in a previous report, we showed that, in Ins-1E and in Min6 beta-cells overexpressing PED/PEA-15, the induction of PKC zeta by glucose was inhibited similar to islets from mice with beta-cell specific overexpression of PED/PEA-15. Moreover, islets from PED/PEA-15 null mice exhibited a two-fold increased activation of PKC zeta by glucose [16]. Both PKC alpha and PKC zeta have been implicated in the intracellular transduction of mitogenic and apoptotic signals [44]–[46], but their role in apoptosis is still unclear. Thus, it is likely that the dysregulation of PKC signalling induced by the overexpression of PED/PEA-15 is responsible for the block of apoptosis in Ins-1E cells. The lack of data addressing the role of the endogenous PED/PEA15 is a limit of this study. Unfortunately, the transfection of specific antisense oligonucleotides resulted in increased toxicity, thereby leading to artifactual results. Our previous results evidenced that selective overexpression of PED/PEA-15 in beta-cells leads to islets hyperplasia and to an increase in beta-cells mass [16]. The results reported in this paper show that these abnormalities are due to an effect of PED/PEA-15 on beta-cells survival mediated by PLD-1. We have previously shown that mice selectively overexpressing PED/PEA-15 in beta-cells feature altered glucose tolerance and impairment of glucose-induced insulin secretion due to impaired expression of the KIR6.2 and SUR1 potassium channel subunits, as observed in transgenic mouse islets and in beta-cell lines stably transfected with PED/PEA-15 cDNA. Thus, we propose that PED/PEA-15 could not only alters beta-cell secretory response to glucose but also increasesthe vitality of the damaged cells. It is possible to speculate that this process might contribute to the gradual exhausting of secretory function due to the reduction of apoptosis of no longer functional beta-cells. In conclusion, a better understanding of the role of PED/PEA-15 in modulating beta-cells survival might contribute also to preserve the normal beta-cells turn over in addition to ameliorate secretory function.

## Supporting Information

S1 Figure Hydrogen peroxide effect on anti- and pro-apoptotic genes expression (dose-response). The expression levels of anti- and pro-apoptotic genes were examined by RT-PCR in Ins-1E_PED/PEA-15_ and in Ins-1E_CTRL_ cells upon incubation with 30–50 µM hydrogen peroxide for 3–8 h. The mRNA amounts of Bcl-xL (**A**), Bcl-xS (**B**) and Bad (**C**) remain unchanged both in Ins-1E_PED/PEA-15_ and in Ins-1E_CTRL_ cells in response to hydrogen peroxide.
(TIF)

S2 Figure Hydrogen peroxide effect on Ins-1E_CTRL_ and Ins-1E_PED/PEA-15_ (dose-response). Ins-1E_CTRL_ and Ins-1E_PED/PEA-15_ were treated for the indicated times (3–8 h) with 30–50 µM hydrogen peroxide and then we evaluated cell viability by sulforhodamine B staining (**A**) and apoptosis measuring the level of DNA fragmentation (**B**). We did not find significant differences both in Ins-1E_CTRL_ and Ins-1E_PED/PEA-15_ upon hydrogen peroxide treatment.
(TIF)

S3 Figure Proliferation curves analysis. We evaluated cell proliferation in Ins-1E_PED/PEA-15_ and in Ins-1E_CTRL_ cells performing proliferation curves analysis. To this aim, Ins-1E_PED/PEA-15_ and Ins-1E_CTRL_ cells were seeded at a density of 2×104 cells in 60 mm dishes. After 24, 48, 72 or 96 h non-adherent cells were removed by gentle washing with PBS whether adherent cells were detached by trypsin treatment and counted using BIO-RAD TC10 automated Cell Counter (Bio-Rad Laboratories, Inc). As shown in S3 Figure, no significant differences were observed in proliferation rate of Ins-1E_PED/PEA-15_ compared to Ins-1E_CTRL_ cells.
(TIF)
