# Supplementary figures and images for: PED/PEA-15 Inhibits Hydrogen Peroxide-Induced Apoptosis in Ins-1E Pancreatic Beta-Cells via PLD-1
Source: PLoS One. 2014 Dec 9;9(12):e113655. doi: 10.1371/journal.pone.0113655 (PMC4260953; doi:10.1371/journal.pone.0113655)

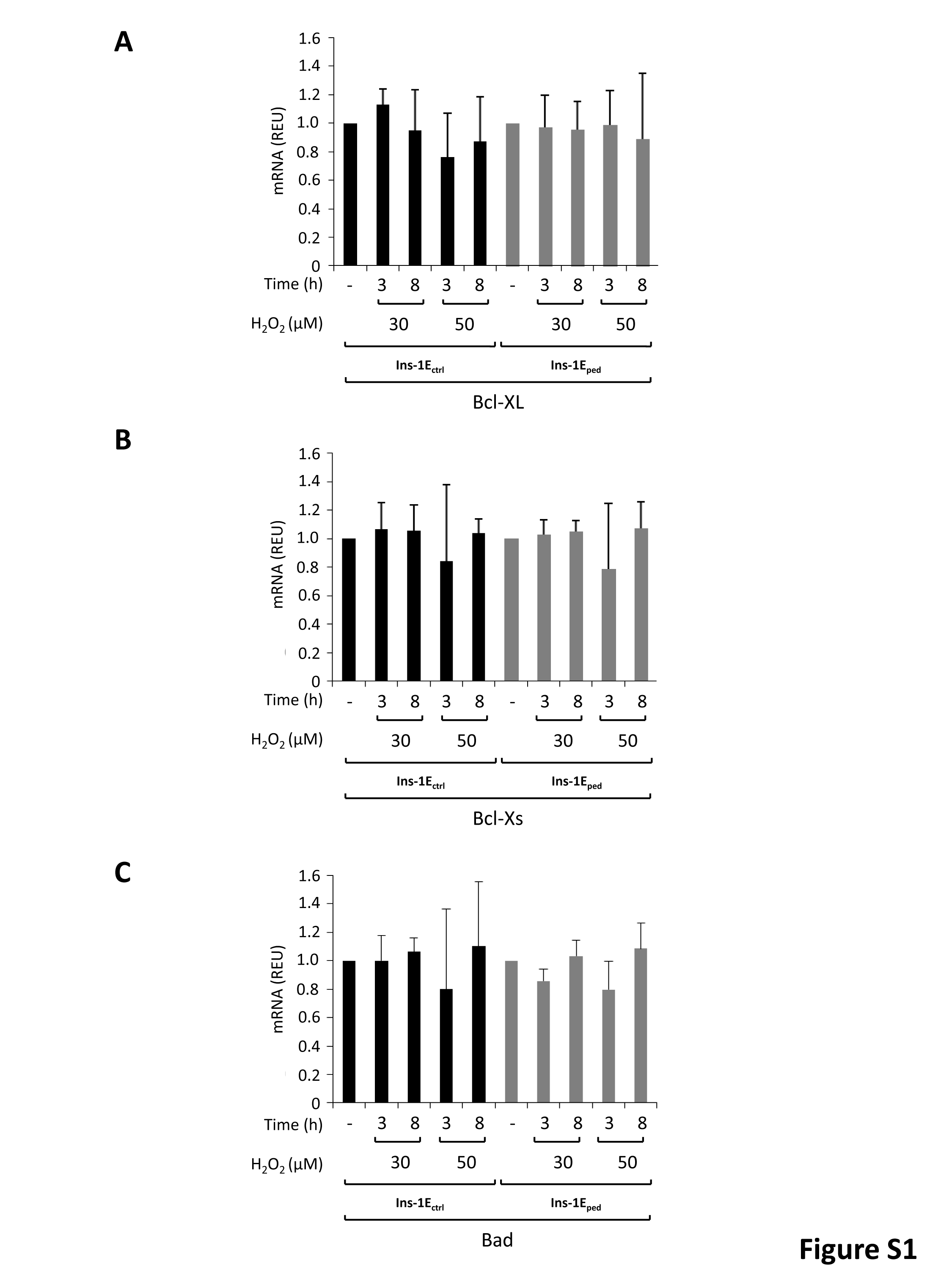

Supplement: S1 Figure — Hydrogen peroxide effect on anti- and pro-apoptotic genes expression (dose-response). The expression levels of anti- and pro-apoptotic genes were examined by RT-PCR in Ins-1EPED/PEA-15 and in Ins-1ECTRL cells upon incubation with 30–50 µM hydrogen peroxide for 3–8 h. The mRNA amounts of Bcl-xL (A), Bcl-xS (B) and Bad (C) remain unchanged both in Ins-1EPED/PEA-15 and in Ins-1ECTRL cells in response to hydrogen peroxide. (TIF) [file pone.0113655.s001.tif]

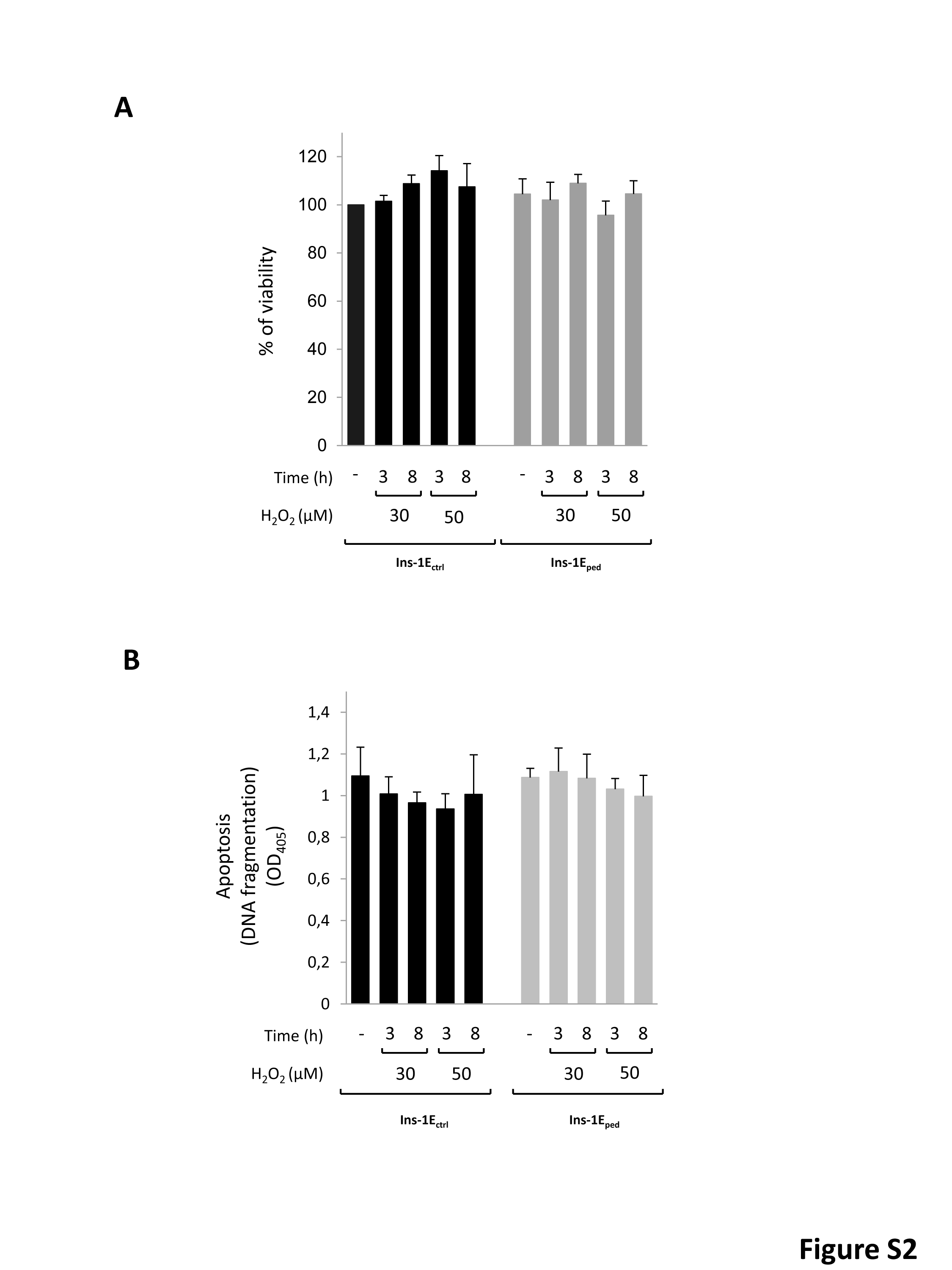

Supplement: S2 Figure — Hydrogen peroxide effect on Ins-1ECTRL and Ins-1EPED/PEA-15 (dose-response). Ins-1ECTRL and Ins-1EPED/PEA-15 were treated for the indicated times (3–8 h) with 30–50 µM hydrogen peroxide and then we evaluated cell viability by sulforhodamine B staining (A) and apoptosis measuring the level of DNA fragmentation (B). We did not find significant differences both in Ins-1ECTRL and Ins-1EPED/PEA-15 upon hydrogen peroxide treatment. (TIF) [file pone.0113655.s002.tif]

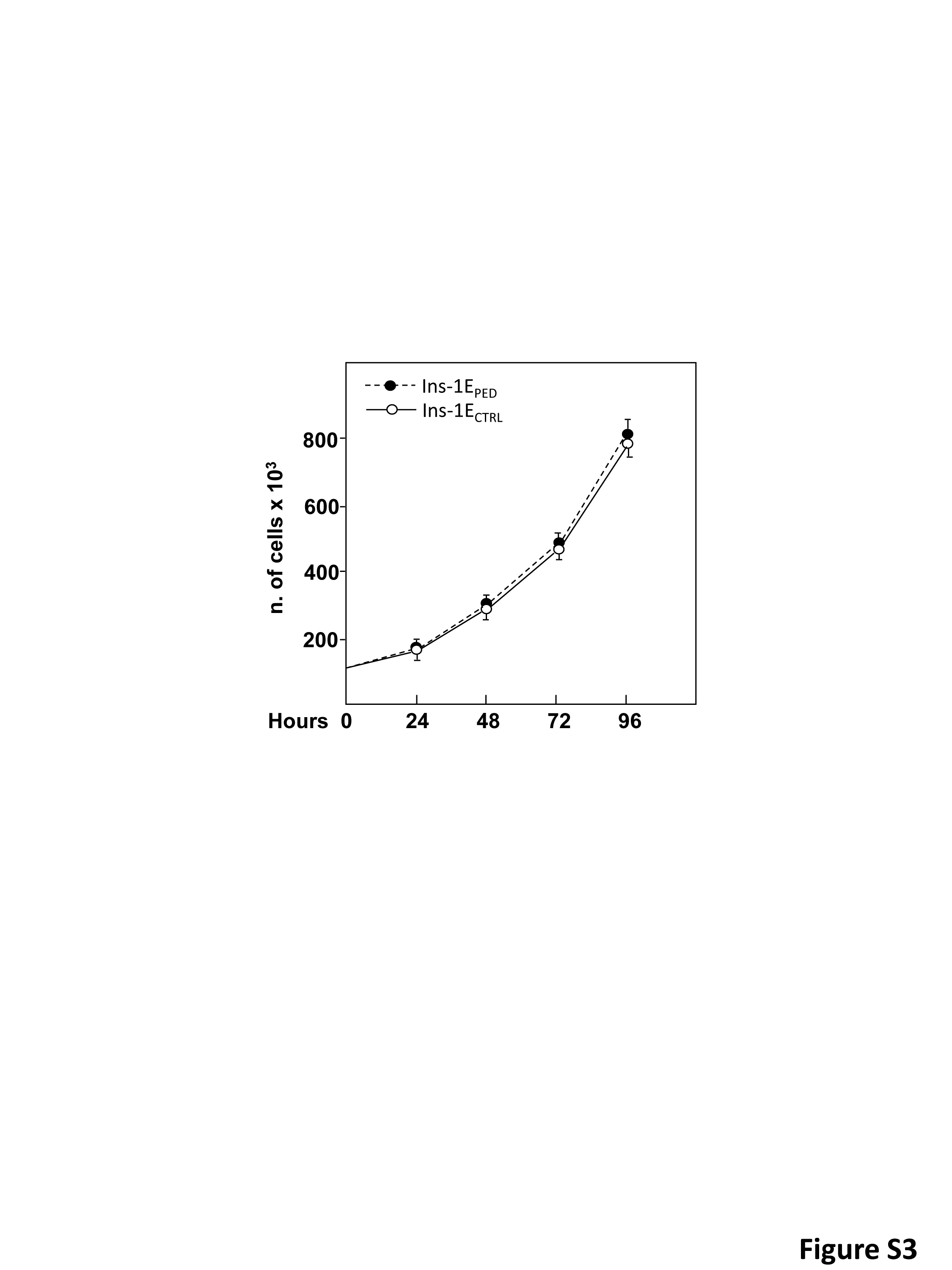

Supplement: S3 Figure — Proliferation curves analysis. We evaluated cell proliferation in Ins-1EPED/PEA-15 and in Ins-1ECTRL cells performing proliferation curves analysis. To this aim, Ins-1EPED/PEA-15 and Ins-1ECTRL cells were seeded at a density of 2×10^4^ cells in 60 mm dishes. After 24, 48, 72 or 96 h non-adherent cells were removed by gentle washing with PBS whether adherent cells were detached by trypsin treatment and counted using BIO-RAD TC10 automated Cell Counter (Bio-Rad Laboratories, Inc). As shown in S3 Figure, no significant differences were observed in proliferation rate of Ins-1EPED/PEA-15 compared to Ins-1ECTRL cells. (TIF) [file pone.0113655.s003.tif]
